# Longitudinal changes of human milk oligosaccharides in Japan and their associations with maternal or infant characteristics

**DOI:** 10.3389/fnut.2026.1850958

**Published:** 2026-05-28

**Authors:** Naoko Higuchi, Kento Sawane, Chisato Hara, Masaya Koshizaka, Midori Yamamoto, Hiroko Takumi, Kenichi Sakurai

**Affiliations:** 1 Ezaki Glico Co., Ltd., Osaka, Japan; 2 Department of Nutrition and Metabolic Medicine, Center for Preventive Medical Sciences, Chiba University, Chiba, Japan; 3 Department of Sustainable Health Science, Center for Preventive Medical Sciences, Chiba University, Chiba, Japan

**Keywords:** breast milk, human milk, human milk oligosaccharides (HMOs), Japanese population, lactation stage, Lewis phenotype, longitudinal study, secretor phenotype

## Abstract

**Introduction:**

Human milk oligosaccharides (HMOs) are the third most abundant solid component in human milk and contribute to infant gut microbiota development, immune maturation, and neurodevelopment. Their composition varies according to genetic factors, particularly maternal Secretor and Lewis phenotypes, as well as lactation stage. However, comprehensive data on longitudinal HMO profiles and genetic phenotypes in Japanese mothers remain limited. This study aimed to characterize longitudinal HMO profiles in Japanese mothers at three lactation stages, classify samples into four Secretor/Lewis groups, and exploratorily evaluate differences associated with maternal and infant characteristics.

**Methods:**

Human milk samples and data were obtained from 270 mothers in the Chiba Study of Mother and Child Health (C-MACH). Samples were collected at colostrum, 1 month, and 4 months postpartum, resulting in 570 samples. Fifteen major HMOs were quantified, and mothers were classified into four milk groups based on Secretor (Se) and Lewis (Le) phenotypes (Se^+^Le^+^, Se^+^Le^−^, Se^−^Le^+^, Se^−^Le^−^). Longitudinal changes in HMO concentrations and group-specific profiles were evaluated. Exploratory analyses assessed associations between HMO concentrations and maternal and infant characteristics across milk groups.

**Results:**

The distribution of milk groups was Se^+^Le^+^ (70.7%), Se^−^Le^+^ (19.6%), Se^+^Le^−^ (7.0%), and Se^−^Le^−^ (2.6%). Total fucosylated HMOs decreased during lactation in Se^+^ mothers but remained stable in Se^−^ mothers. HMOs characteristic of Se^+^ mothers, including 2′-fucosyllactose, declined over time, whereas 3-fucosyllactose (3-FL) increased in all groups and was consistently higher in Le^+^ mothers. Acetylated and sialylated HMOs generally declined, with acetylated HMOs being consistently higher in Se^−^Le^−^ mothers. No significant associations between maternal or infant characteristics and HMO concentrations were detected in the Se^+^Le^+^ group; however, in the Se^−^Le^+^ group, 3-FL concentrations in colostrum were lower in multiparous mothers.

**Conclusions:**

This study provides the first comprehensive description of longitudinal HMO profiles in Japanese mothers stratified according to Secretor/Lewis groups. Distinct temporal patterns were identified across milk groups, and exploratory findings suggest potential group-specific associations between parity and certain HMOs. These results contribute to the understanding of interindividual variability in HMO synthesis and provide foundational data for future mechanistic and population-based studies.

## 1 Introduction

Human milk is widely recognized as the optimal source of nutrition for infants (1). In addition to providing essential nutrients that support physical growth, breastfeeding also contributes to neurodevelopment and immune system maturation, leading to lifelong health (1).

Human milk contains various macronutrients (e.g., carbohydrates, fats, and proteins) that play important roles in infant growth (2). Human milk oligosaccharides (HMOs) are the third most abundant solid component of human milk after lactose and fat (3). HMOs consist of a lactose core that is further lengthened with monosaccharides, such as galactose, fucose, and sialic acid (4). To date, > 200 HMO molecules have been identified in human milk, with approximately 50 molecules representing the majority of its total composition (3). Most HMOs are resistant to digestion in the small intestine and reach the colon intact (5), where they exert several biological functions. For example, 2′-fucosyllactose (2′-FL) promotes the growth of bifidobacteria and establishment of a beneficial gut microbiota in infants (6–8) and contributes to the development of the infant immune system (9). In addition, sialylated HMOs, such as 3′-sialyllactose (3′-SL) and 6′-sialyllactose (6′-SL), are associated with infants' neurodevelopment (10, 11), potentially mediated by the production of sialic acid through microbial degradation (12). Thus, each HMO molecule exerts different biological functions, and the HMO composition in human milk is associated with infant growth and development.

The HMO composition of human milk is influenced by genetic factors (13, 14). Milk from mothers with active α1,2-fucosyltransferase (FUT2) expression (“secretors,” Se^+^) contains high levels of α1,2-fucosylated HMOs, such as 2′-FL and lacto-N-fucopentaose I (LNFP I), whereas these compounds are nearly absent in the milk of mothers lacking FUT2 expression (“non-secretors,” Se^−^) (14, 15). α1,3-fucosyltransferase (FUT3) is another genetic determinant that defines the Lewis phenotype (16). Mothers who express FUT3 (“Lewis-positive,” Le^+^) produce α1,3/4-fucosylated HMOs, such as LNFP II, whereas these structures are absent in milk from mothers who lack FUT3 expression (“Lewis-negative,” Le^−^) (15). Recent studies have classified lactating mothers into four groups based on combined Secretor and Lewis phenotypes (Se^+^Le^+^, Se^−^Le^+^, Se^+^Le^−^, and Se^−^Le^−^) using the HMO concentrations in human milk (17). Although most reports have consistently described the highest prevalence for Se^+^Le^+^, followed by Se^−^Le^+^, Se^+^Le^−^, and Se^−^Le^−^, the proportions differ across countries and regions (18, 19).

In addition to genetic factors, non-genetic factors contribute to variations in HMO concentrations (20–24). The lactation stage is one influential factor: the concentrations of most HMOs are the highest in colostrum and gradually decrease during lactation, whereas the concentrations of specific HMOs, such as 3-fucosyllactose (3-FL), increase over time (21, 22, 25). Maternal and infant characteristics—such as pre-pregnancy body mass index (BMI), parity, delivery mode, infant sex, maternal age, and breastfeeding status—are also associated with HMO concentration; however, the results across studies remain inconsistent (19, 20, 23, 24, 26, 27). These inconsistencies are likely due to differences in maternal and infant characteristics across populations or to inadequate consideration of genetic factors such as maternal Secretor and Lewis phenotypes. Therefore, it is important to evaluate these associations within each country or region stratified according to the maternal Secretor/Lewis phenotype to comprehensively understand the association between maternal or infant characteristics and HMO compositions.

Compared with Western countries, information on HMOs in East and Southeast Asian populations remains limited (28–33), specifically in Japanese populations (34–38). Comprehensive data on Secretor/Lewis group distribution, longitudinal HMO patterns, and related maternal or infant factors are unavailable in the Japanese population. To address this, we aimed to analyze HMOs in human milk collected from Japanese mothers at three lactation stages (colostrum, 1 month, and 4 months postpartum). Based on the data, we classified mothers into four milk groups according to their Secretor and Lewis phenotypes and examined differences in HMO profiles and longitudinal changes. In addition, we examined the differences in HMO concentrations stratified according to maternal and infant characteristics across milk groups.

## 2 Methods

### 2.1 Study design

The study participants were selected from mother–child pairs recruited during different enrollment periods of the Chiba Study of Mother and Child Health (C-MACH) phases 1 (39) and 2 (40). The study protocol was approved by the Biomedical Research Ethics Committee of the Graduate School of Medicine, Chiba University (protocol code: 1199; approval date: June 1, 2022), and written informed consent was obtained from all participating mothers for themselves and their children. This study was conducted in accordance with the Strengthening the Reporting of Observational Studies in Epidemiology (STROBE) guidelines.

**Figure 1:**
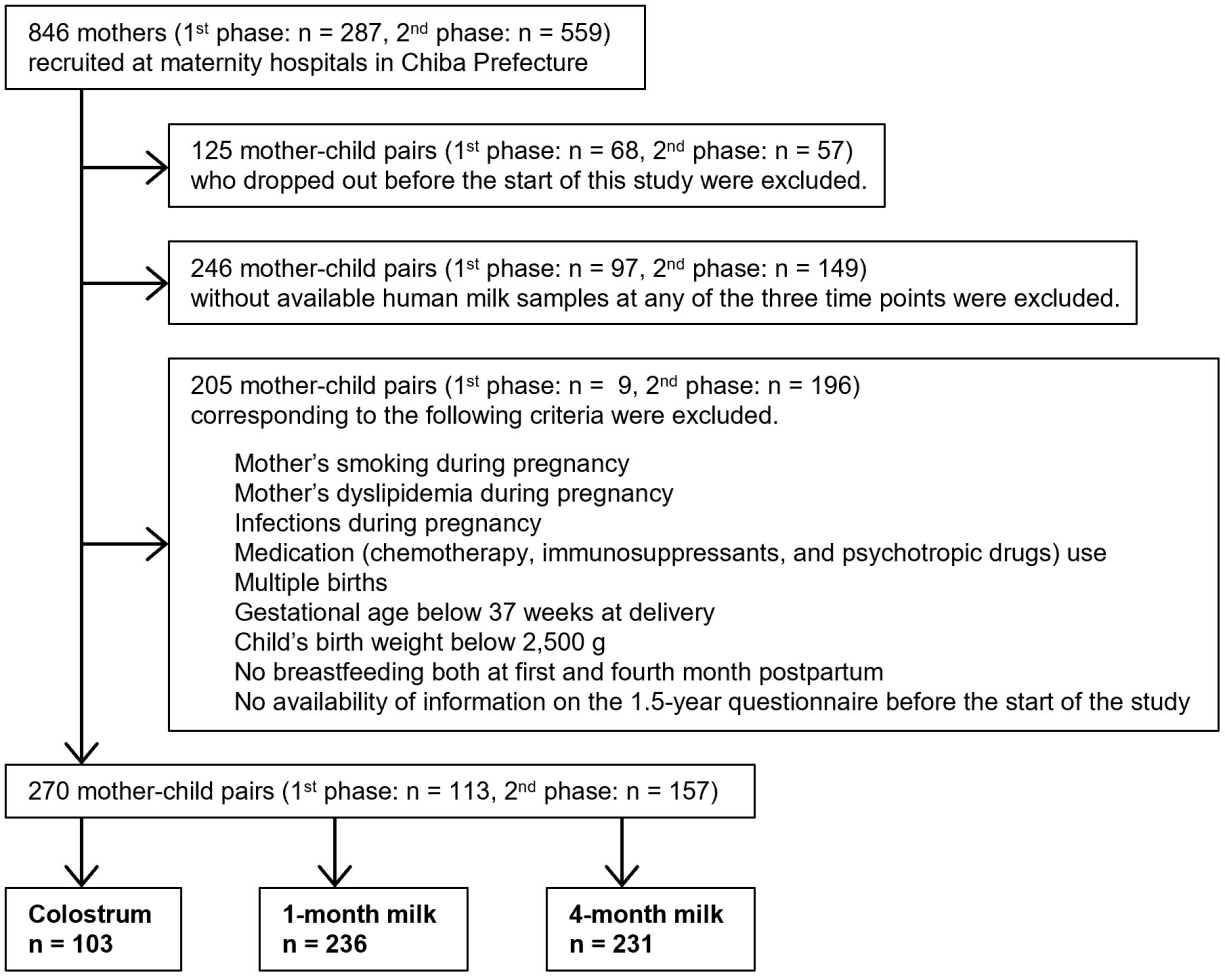
Flowchart for the study population.

Study participants were selected from among 846 mother–child pairs registered with the C-MACH in Chiba Prefecture. After applying the following exclusion criteria, 270 mother–child pairs were included in the final analysis. The exclusion criteria were as follows: withdrawal from the C-MACH; without available human milk samples at any of the three lactation stages (colostrum, 1 month, and 4 months postpartum); maternal smoking during pregnancy; maternal dyslipidemia during pregnancy; maternal infections during pregnancy that could cause fetal or neonatal infection (41); use of medications including anticancer drugs, immunosuppressants, or psychotropic drugs; multiple births; gestational age < 37 weeks at delivery; infant birth weight < 2,500 g; no breastfeeding at both 1 and 4 months postpartum; and unavailability of the 1.5-year questionnaire at the beginning of this study. Among the included participants, human milk samples were available as follows: colostrum (*n* = 103), 1-month milk (*n* = 236), and 4-month milk (*n* = 231) (Figure 1). The smaller number of colostrum samples reflects the study design: colostrum was collected only in the phase 1 protocol.

### 2.2 Questionnaires and medical records

Maternal information was obtained using a combination of self-administered questionnaires and medical records. Mothers completed the questionnaires at 12 and 32 weeks of gestation and again at 1, 4, 10, and 18 months postpartum. Pre-pregnancy BMI was calculated from the self-reported height and weight provided in the questionnaire at 12 weeks of gestation. Data on maternal age, smoking history, dyslipidemia, pregnancy-related infections, and medication use were collected from the questionnaires administered during pregnancy. Information on parity (presence and number of older siblings) and breastfeeding practices was derived from questionnaires collected until 4 months after delivery. Data on gestational age at birth, mode of delivery, infant birth weight, and infant sex were extracted from medical records at delivery.

### 2.3 Human milk collection

In the phase 1 study, human milk samples were collected at three time points: during postpartum hospitalization (colostrum) and at 1 and 4 months postpartum. In the phase 2 study, samples were collected at 1 and 4 months postpartum. Human milk samples were collected by the mothers using manual expression into the designated collection tubes. Colostrum was obtained in 2-mL tubes, with up to 6 mL collected in total, and was immediately stored at −80 °C at the hospital before transport. For milk collected at 1 and 4 months postpartum (hereafter referred to as “1-month milk” and “4-month milk”), the mothers were instructed to discard a small amount of foremilk and then express up to 15 mL into a 50-mL centrifuge tube. The samples were stored in a home freezer for at least 12 h before transportation. All samples were transferred to Chiba University under frozen conditions and stored at −80 °C upon arrival. Before further preprocessing, the samples were thawed once for dispensing and then returned to −80 °C for storage until defatting and subsequent analyses, as described below.

### 2.4 Preparation of human milk for HMO analysis

Human milk samples were prepared as previously described with modifications (35, 42). Human milk samples were spiked with an internal standard solution of xylose. The samples were centrifuged at 5,000 × g for 15 min at 4 °C to remove the fat layer. The resulting supernatant was mixed with ethanol at a 1:3 ratio and stored at −20 °C, followed by centrifugation at 14,000 × g for 10 min at 4 °C to precipitate proteins. The resulting supernatant was applied to an Amicon Ultra-0.5 mL centrifugal filter (10 kDa; cat# UFC5010, Merck Millipore, Billerica, MA, USA), which was pre-washed with 4.5 mM ammonium formate prior to sample loading. After centrifugation at 14,000 × g for 30 min at 4 °C, the filter was washed again with 4.5 mM ammonium formate and centrifuged under the same conditions. The filtrate was diluted with pure water and used for analysis.

### 2.5 Liquid chromatography–tandem mass spectrometry analysis of HMOs

HMOs were analyzed using an LCMS-8060 system (Shimadzu, Kyoto, Japan) coupled with a Nexera X2 ultra-high-performance liquid chromatography (Shimadzu), following published methods with modifications (43–45). Chromatographic separation was performed on an ACQUITY UPLC BEH Amide column (2.1 mm × 100 mm, 1.7 μm; Waters, Milford, MA, USA) at a flow rate of 0.3 mL/min. The mobile phase was a binary solvent system consisting of (A) 10 mM ammonium formate in water and (B) acetonitrile. The gradient program was as follows: 85% B at 0 min, 58% B at 20 min, 40% B at 20.1 min, maintained at 40% B until 25 min, then returned to 85% B at 25.1 min, and held until 35 min.

**Figure 2:**
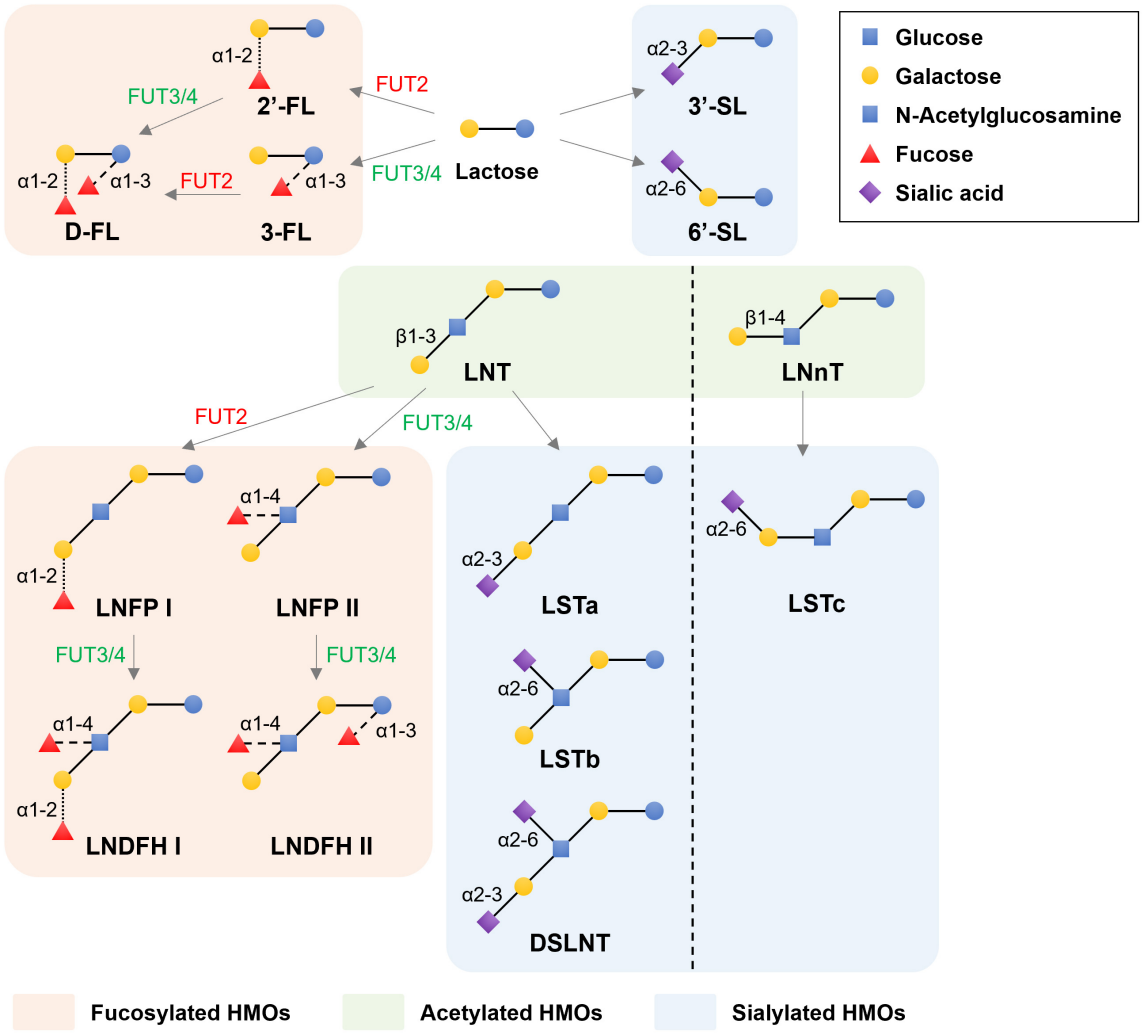
Human milk oligosaccharide structures analyzed in this study. Fucosylation at the α1–2 position (dotted lines) is mediated by α1,2-fucosyltransferase (FUT2), whereas fucosylation at the α1–3 or α1–4 position (dashed lines) is catalyzed by α1,3/4-fucosyltransferases (FUT3/4). 2′-FL, 2′-fucosyllactose; 3-FL, 3-fucosyllactose; 3′-SL, 3′-sialyllactose; 6′-SL, 6′-sialyllactose; D-FL, difucosyllactose; DSLNT, disialyllacto-N-tetraose; FUT2, α1,2-fucosyltransferase; FUT3/4, α1,3/4-fucosyltransferase; HMO, human milk oligosaccharide; LNDFH I/II, lacto-N-difucohexaose 1/2; LNFP I/II, lacto-N-fucopentaose 1/2; LNnT, lacto-N-neotetraose; LNT, lacto-N-tetraose; LSTa/b/c, sialyl-lacto-N-tetraose a/b/c.

Quantification was performed using authentic standards for 15 HMO species. Detection was performed in negative ion mode, and compound-specific parameters were optimized for each analyte. Calibration curves were constructed using serial dilutions of a mixed standard solution containing all 15 HMOs (2′-FL, 3-fucosyllactose [3-FL], difucosyllactose [D-FL], lacto-N-fucopentaose I [LNFP I], LNFP II, lacto-N-difucohexaose I [LNDFH I], lacto-N-difucohexaose II [LNDFH II], lacto-N-tetraose [LNT], lacto-N-neotetraose [LNnT], 3′-SL, 6′-SL, sialyl-lacto-N-tetraose a [LSTa], sialyl-lacto-N-tetraose b [LSTb], sialyl-lacto-N-tetraose c [LSTc], and disialyllacto-N-tetraose [DSLNT]) (Figure 2) and 2.5 pg/μL xylose as an internal standard, with 1/x weighting applied during regression. Calibration accuracy was confirmed using the coefficient of determination (R^2^ ≥ 0.98) (34). Detailed information on the standards, detection conditions, and calibration curves is provided in Supplementary Table S1. Concentrations were calculated using the internal standard method with xylose, and calibration curves were established. For HMO concentrations below the limit of quantification (LOQ), values were imputed as one-half of the LOQ prior to statistical analyses.

### 2.6 Maternal Secretor and Lewis phenotypes

Both maternal Secretor (Se) and Lewis (Le) phenotypes were determined using HMO concentrations in milk samples, based on the classification criteria reported previously (19, 24, 34). Specifically, milk samples with concentrations of 2′-FL > 100 mg/L and LNFP II > 25 mg/L were assigned to the Se^+^Le^+^ group, milk samples with concentrations of 2′-FL > 100 mg/L and LNFP-II < 25 mg/L were assigned to the Se^+^Le^−^ group, milk samples with concentrations of 2′-FL < 100 mg/L and LNFP II > 25 mg/L were assigned to the Se^−^Le^+^ group, and milk samples with concentrations of 2′-FL < 100 mg/L and LNFP-II < 25 mg/L were assigned to the Se^−^Le^−^ group. The same criteria were applied at all time points to ensure that each mother remained in the same group for all measurements.

### 2.7 Statistical analyses

Participant characteristics are summarized as means and standard deviations (SDs) for continuous variables and as counts and percentages for categorical variables. Total concentrations were calculated for specific HMO categories: fucosylated HMOs (including 2′-FL, 3-FL, D-FL, LNFP I, LNFP II, LNDFH I, and LNDFH II), acetylated HMOs (LNT and LNnT), and sialylated HMOs (3′-SL, 6′-SL, LSTa, LSTb, LSTc, and DSLNT) (Figure 2). Longitudinal changes in HMO concentrations were analyzed in all mothers, and in a subset of mothers with measurements available at all time points (*n* = 79). HMO concentrations were summarized for the four milk groups as mean and 95% confidence intervals or medians and interquartile ranges.

For the association analysis between maternal and infant characteristics and HMO concentrations, the characteristics were converted to categorical variables as follows: maternal age at delivery (≥ 35 vs. < 35 years), pre-pregnancy BMI (< 18.5 vs. 18.5–25 kg/m^2^ and ≥ 25 vs. 18.5–25 kg/m^2^), parity (multiparous vs. primiparous), delivery mode (cesarean vs. vaginal), infant sex (male vs. female), and breastfeeding status at 1 and 4 months (mixed feeding vs. exclusive breastfeeding). To calculate fold changes, the mean HMO concentrations of each group were divided by that of the reference group, defined as the latter category listed, and the resulting ratios were log_2_-transformed. Group differences between the two categories of each maternal and infant characteristic were evaluated using Welch's *t*-test with false discovery rate adjustment (Benjamini–Hochberg correction) on log_2_-transformed HMO concentrations for skewed distributions. Adjusted *p*-values < 0.05 were considered statistically significant. Groups consisting of five or fewer participants were excluded from the analysis.

Statistical analyses and data visualization were performed using the R software (version 4.2.2; R Foundation for Statistical Computing, Vienna, Austria).

## 3 Results

### 3.1 Characteristics of the study population

The participant characteristics for the entire study population (*n* = 270) are summarized in Table 1. The maternal age at recruitment was 33.3 years (SD, 4.1). A total of 44.4% of the mothers had a female infant, 38.9% were primipara, and 18.5% gave birth by cesarean section. Maternal pre-pregnancy BMI was as follows: 12.2% were < 18.5 kg/m^2^, 75.9% were between 18.5–25 kg/m^2^, and 10.7% were > 25 kg/m^2^. The characteristics of both the phase 1 and 2 studies are provided in Supplementary Table S2.

**Table 1:** Characteristics of the study population.

Variables	Overall (*n* = 270)
Maternal characteristics	
Age at recruitment (years), mean (SD)	33.3 (4.1)
Pre-pregnancy BMI (kg/m^2^), *n* (%)	
< 18.5	33 (12.2%)
18.5–25	205 (75.9%)
≥25	29 (10.7%)
NA	3 (1.1%)
Parity, *n* (%)	
Primipara	105 (38.9%)
Multipara	165 (61.1%)
Child characteristics	
Gestational age at birth (weeks), mean (SD)	39.3 (1.0)
Birth weight (g), mean (SD)	3,088 (320)
Sex, *n* (%)	
Female	120 (44.4%)
Male	150 (55.6%)
Delivery mode, *n* (%)	
Vaginal delivery	217 (80.4%)
Cesarean section	50 (18.5%)
NA	3 (1.1%)
Breastfeeding status at 1 month of age, *n* (%)	
Exclusive breastfeeding	72 (26.7%)
Mixed feeding	198 (73.3%)
Breastfeeding status at 4 months of age, *n* (%)	
Exclusive breastfeeding	111 (41.1%)
Mixed feeding	159 (58.9%)
HMO milk group, *n* (%)	
Se^+^Le^+^	191 (70.7%)
Se^+^Le^−^	19 (7.0%)
Se^−^Le^+^	53 (19.6%)
Se^−^Le^−^	7 (2.6%)

BMI, body mass index; HMO, human milk oligosaccharide; Le^+^/Le^−^, Lewis-positive/Lewis-negative; NA, not available; SD, standard deviation; Se^+^/Se^−^, Secretor-positive/Secretor-negative. Data are presented as means (SDs) for continuous variables and n (%) for categorical variables.

### 3.2 Longitudinal changes in HMO concentrations across the Secretor/Lewis groups

Human milk samples were collected from a total of 270 mothers: colostrum (*n* = 103), 1-month milk (*n* = 236), and 4-month milk (*n* = 231) (Figure 1). The distribution of four milk groups was as follows: Se^+^Le^+^ (*n* = 191, 70.7%), Se^+^Le^−^ (*n* = 19, 7.0%), Se^−^Le^+^ (*n* = 53, 19.6%), and Se^−^Le^−^ (*n* = 7, 2.6%) (Table 1). The distributions of 2′-FL and LNFP II concentrations at each time point, which were used as indicators for group classification, are shown in Supplementary Figure S1.

**Figure 3:**
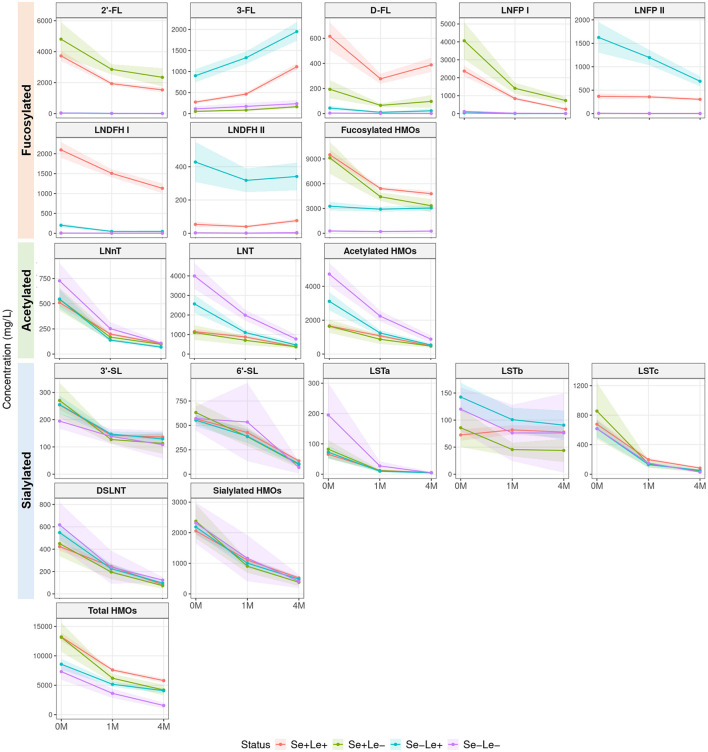
Longitudinal changes of human milk oligosaccharide concentrations according to milk groups. Colostrum (0M, *n* = 103), 1-month milk (1M, *n* = 236), and 4-month milk (4M, *n* = 231). Colored vertical bars on the left indicate the groups based on HMO structures (fucosylated, acetylated, and sialylated). Concentrations are described by mg/L. Each dot represents the mean, and shaded areas represent the 95% confidence interval. All statistical results are shown in Supplementary Tables S3–S5. 0M, colostrum; 1M, 1-month milk; 2′-FL, 2′-fucosyllactose; 3-FL, 3-fucosyllactose; 3′-SL, 3′-sialyllactose; 4M, 4-month milk; 6′-SL, 6′-sialyllactose; D-FL, difucosyllactose; DSLNT, disialyllacto-N-tetraose; HMO, human milk oligosaccharide; Le^+^/Le^−^, Lewis-positive/Lewis-negative; LNDFH I/II, lacto-N-difucohexaose 1/2; LNFP I/II, lacto-N-fucopentaose 1/2; LNnT, lacto-N-neotetraose; LNT, lacto-N-tetraose; LSTa/b/c, sialyl-lacto-N-tetraose a/b/c; Se^+^/Se^−^, Secretor-positive/Secretor-negative.

Longitudinal changes in individual HMO species and total HMOs are shown in Figure 3. Total fucosylated HMO concentrations decreased across lactation stages in Se^+^ mothers, whereas they were stable in Se^−^ mothers. The concentrations of 2′-FL and LNFP I, which are synthesized in Se^+^ mothers, decreased across lactation stages and were consistently higher in the Se^+^Le^−^ group than in the Se^+^Le^+^ group at all time points. In contrast, the concentrations of 3-FL increased over time in all groups and were consistently higher in the Le^+^ groups. The concentrations of D-FL (synthesized in Se^+^ mothers) and LNDFH II (synthesized in Le^+^ mothers), which, like 3-FL, contain the α1-3 linkage, decreased from colostrum to 1-month milk but remained stable or showed slight increases from 1 month to 4 months. The concentrations of LNFP II (synthesized in Le^+^ mothers) decreased over time in Se^−^Le^+^ but remained relatively constant in Se^+^Le^+^.

The concentrations of acetylated HMOs, including LNnT and LNT, decreased across lactation stages. Total acetylated HMOs and LNT concentrations were higher in the colostrum of Se^−^ mothers, with concentrations being consistently highest in the Se^−^Le^−^ group at all time points.

The concentrations of the total sialylated HMOs, as well as 3′-SL, 6′-SL, LSTa, LSTc, and DSLNT, showed longitudinal decreases, which were consistent among all milk groups. LSTc was the most abundant sialylated HMO in colostrum, but not at 1 month or 4 months.

The detailed summary statistics are provided in Supplementary Tables S3–S5. The overall longitudinal patterns were similar when analyses were stratified according to the mothers who provided milk to all three samples (*n* = 79) (Supplementary Figure S2, Supplementary Tables S6–S8).

### 3.3 Associations between HMO concentrations and maternal or infant characteristics

**Figure 4:**
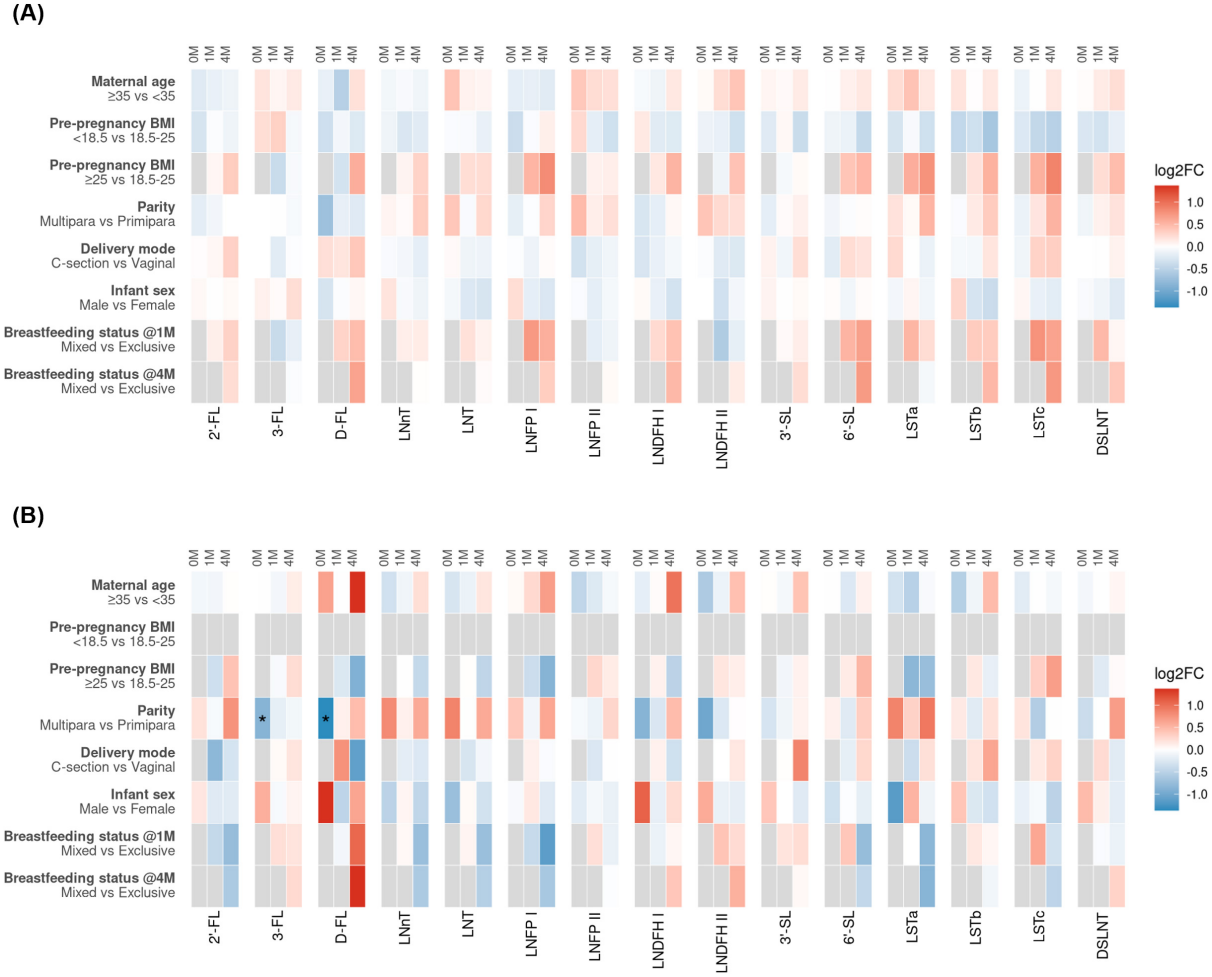
Associations between human milk oligosaccharide concentrations and maternal or infant characteristics, **(A)** Se^+^Le^+^, and **(B)** Se^−^Le^+^. Log_2_-transformed fold changes (log2FC) were calculated from group means of the HMO concentrations. In each comparison indicated as “A vs. B,” the reference group corresponds to the group listed on the right side (“B”). Colors indicate the direction of the log2FC value (blue, negative and red, positive). Statistical significances were evaluated using Welch's *t*-test applied to the log_2_-transformed concentration data, followed by false discovery rate adjustment. *Adjusted *p*-value < 0.05. All statistical results are shown in Supplementary Tables S9 and S10. 0M, colostrum; 1M, 1-month milk; 2′-FL, 2′-fucosyllactose; 3-FL, 3-fucosyllactose; 3′-SL, 3′-sialyllactose; 4M, 4-month milk; 6′-SL, 6′-sialyllactose; BMI, body mass index; C-section, cesarean section; D-FL, difucosyllactose; DSLNT, disialyllacto-N-tetraose; Le^+^, Lewis-positive; LNDFH I/II, lacto-N-difucohexaose 1/2; LNFP I/II, lacto-N-fucopentaose 1/2; LNnT, lacto-N-neotetraose; LNT, lacto-N-tetraose; log2FC, log_2_ fold changes; LSTa/b/c, sialyl-lacto-N-tetraose a/b/c; Se^+^/Se^−^, Secretor-positive/Secretor-negative.

Owing to limited sample sizes in several subgroups, comparisons were conducted only within the Se^+^Le^+^ and Se^−^Le^+^ groups. In the Se^+^Le^+^ group, no significant associations were observed (Figure 4A and Supplementary Table S9). In contrast, in the Se^−^Le^+^ group, the colostrum of multiparous mothers had significantly lower concentrations of 3-FL and D-FL (Figure 4B and Supplementary Table S10). Although not statistically significant, a similar trend for D-FL was also observed in the Se^+^Le^+^ group.

## 4 Discussion

In this study, human milk samples collected from Japanese mothers at three lactation stages—colostrum, 1-month milk, and 4-month milk—were analyzed to quantify 15 major HMOs. Based on these measurements, the mothers were classified into four groups according to their Secretor and Lewis phenotypes, and longitudinal changes in HMO concentrations were characterized within each group. To the best of our knowledge, this is the first study in the Japanese population to apply the four-group Secretor/Lewis classification and describe group-specific temporal patterns of HMO concentrations across lactation stages. In addition, exploratory association analyses revealed that, in the Se^−^Le^+^ group, several HMOs were associated with specific maternal and infant characteristics.

### 4.1 Secretor/Lewis phenotype in the Japanese population

In this study, the Se^+^Le^+^ group was the most prevalent (70.7%), followed by Se^−^Le^+^ (19.6%), Se^+^Le^−^ (7.0%), and Se^−^Le^−^ (2.6%). The proportion of secretor mothers in this study (sum of Se^+^Le^+^ and Se^+^Le^−^, 77.7%) was consistent with reports from Japanese populations (34, 35). Studies examining the distribution of the four Secretor/Lewis groups in China (29–31) and Western countries (15, 19, 23, 24, 46) have shown that their proportions are broadly similar across regions. In this study, we described the distribution of the four milk groups in the Japanese population, and the observed proportions fell within the range reported in these earlier studies.

### 4.2 Longitudinal changes in HMO concentrations

In this study, the longitudinal changes in HMO concentrations in the Japanese population were largely consistent with those described in published studies (e.g., decreases in multiple HMOs, including 2′-FL and LNFP I, and an increase in 3-FL) (19, 22, 25). In addition, we obtained several insights into the dynamics of FUT2- and FUT3-related enzymatic activities.

First, the total concentration of fucosylated HMOs was highest in colostrum among secretor mothers (Se^+^Le^+^, Se^+^Le^−^) and showed a clear decrease over time. Moreover, within the secretor mothers, the Se^+^Le^−^ group—considered to have a stronger dependence on FUT2 for HMO fucosylation—exhibited an even greater decline. These observations suggest that FUT2 activity is the highest in colostrum and subsequently decreases during lactation.

In contrast, FUT3 activity appeared to remain relatively stable during lactation, based on the observed HMO concentration patterns. This interpretation is supported by the finding that the total amount of fucosylated HMOs in the Se^−^Le^+^ group, which is considered more dependent on FUT3 for fucosylated HMO synthesis than other groups, showed little temporal change. Additionally, within this group, the opposing temporal patterns—the increase in 3-FL concentration and the decline in LNFP II concentration—were the most pronounced. These patterns may reflect the reaction dynamics driven by changes in the concentration of LNT, a substrate for FUT3. LNT concentrations were highest in the Se^−^Le^−^ group, likely because low enzymatic activities of both FUT2 and FUT3 limit its utilization as a substrate for fucosylation. These interpretations agree with the findings of a published study showing that individual HMO concentrations depend on the interplay between FUT2 and FUT3 activities and the availability of relevant substrates (13).

The total concentration of sialylated HMOs decreased during lactation in all groups, and the inter-group differences were smaller than those observed for fucosylated or acetylated HMOs. As with fucosylated oligosaccharides, longitudinal changes in enzyme activity or substrate availability are plausible contributors; however, individual variation cannot be attributed to FUT2 or FUT3 activity. Moreover, LSTc was the most abundant sialylated HMO in colostrum, but not in mature milk, indicating its potential role in the early postnatal period. However, its structure-specific functions have not been elucidated.

### 4.3 Association with maternal or infant characteristics

In the Se^+^Le^+^ group, no significant associations were detected between the HMO concentrations and maternal or infant characteristics. In contrast, in the Se^−^Le^+^ group, 3-FL and D-FL concentrations in colostrum differed according to maternal parity. Some studies conducted similar association analyses adjusted for milk groups in their statistical models (19, 23, 24), whereas others performed stratified analyses based only on the Secretor phenotype (28, 47, 48). However, the large differences in HMO profiles among the four milk groups suggest that stratification according to milk groups is also important for identifying group-specific associations, as demonstrated in an earlier study (49) and our analysis. Nevertheless, both studies had limited sample sizes; therefore, larger studies are required to further investigate these associations.

Considering the lower 3-FL concentration observed in the colostrum of multiparous mothers in the Se^−^Le^+^ group (with D-FL also showing lower concentration, although its discussion is omitted because synthesis in Se^−^ groups is minimal), some studies have documented similar associations in mature milk (23, 26). These studies have described higher LNT and LNnT concentrations among multiparous mothers, a pattern that, although not statistically significant, was also apparent in both the Se^+^Le^+^ and Se^−^Le^+^ groups in our study. Although causal mechanisms remain unknown, these observations suggest that parity modulates the balance between enzymatic activity and substrate availability during HMO synthesis. Further studies are required to identify the biological processes underlying this association.

### 4.4 Strengths

This is the first study in Japan to provide a detailed description of HMO concentrations from colostrum to mature milk and to examine longitudinal changes across lactation stages. Second, this study is the first to compare HMO profiles in Japanese mothers according to both Secretor and Lewis phenotypes, providing novel insights into genetic influences on milk composition in this population. Finally, these findings offer valuable baseline data for global comparisons of the HMO composition across populations, contributing to a broader understanding of ethnic and regional variability in lactation biology.

### 4.5 Limitations

This study has some limitations. First, the sample size was relatively small, which prevented us from analyzing associations between HMO concentrations and maternal or infant characteristics in Se^+^Le^−^ and Se^−^Le^−^ groups. Second, the number of participants from whom colostrum samples were collected was relatively small, which reduced the robustness of the analysis across the time points. Third, the study population was recruited from a limited geographic area in Chiba Prefecture, which limits the generalizability of our findings. However, studies conducted in other Japanese regions have reported similar distributions of Secretor status (34, 35). Fourth, we quantified 15 HMO species, whereas other studies have analyzed more molecules. The measurement of additional species can provide deeper insights into compositional diversity and functional implications. Fifth, because the human milk samples were collected by the mothers themselves, home-based collection and storage conditions were not strictly standardized. Finally, given the descriptive design and univariate analyses, potential confounding factors were not accounted for. These results should be considered hypothesis-generating for future analytical investigations.

This study provides the first comprehensive description of longitudinal HMO profiles in Japanese mothers stratified according to both Secretor and Lewis phenotypes. These findings highlight the distinct temporal patterns across milk groups and suggest potential group-specific associations between parity and certain HMOs. These results contribute to the understanding of interindividual variability in HMO synthesis and provide foundational data for future mechanistic and population-based studies.

## Data Availability

The original contributions presented in the study are included in the article/Supplementary material, further inquiries can be directed to the corresponding author.
