# Supplementary material for: Longitudinal changes of human milk oligosaccharides in Japan and their associations with maternal or infant characteristics
Source: Front Nutr. 2026 May 28;13:1850958. doi: 10.3389/fnut.2026.1850958 (PMC13254561; doi:10.3389/fnut.2026.1850958)
Supplement: Supplementary file 1 [file Image_1.pdf]

Supplementary Material

Supplementary Figures

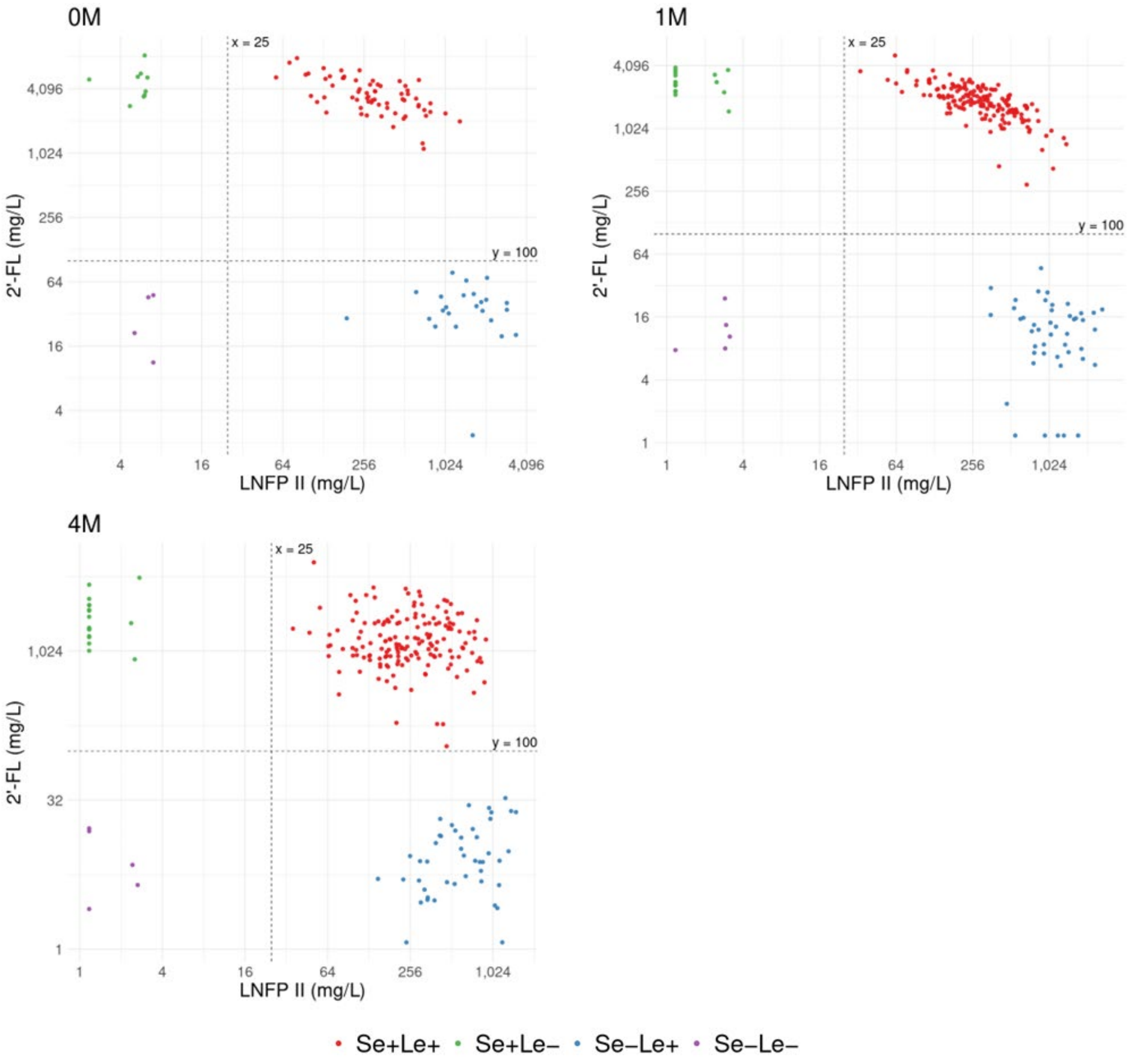

Supplementary Figure 1. Distributions of 2'-fucosyllactose (2'-FL) and lacto-N-fucopentaose 2 (LNFP II) concentrations in colostrum (0M, n = 103), 1-month milk (1M, n = 236), and 4-month milk (4M, n = 231). Each dot represents an individual mother, and colors indicate the four milk groups. Both axes are $\log_{10}$-transformed (labels indicate original mg/L values). The dashed lines represent the cut-off values used for milk-group classification: LNFP II = 25 mg/L (x = 25) and 2'-FL = 100 mg/L (y = 100).

Abbreviations: 0M, colostrum; 1M, 1-month milk; 2'-FL, 2'-fucosyllactose; 4M, 4-month milk; Le^+/Le^-, Lewis-positive/Lewis-negative; LNFP II, lacto-N-fucopentaose II; Se^+/Se^-, Secretor-positive/Secretor-negative.

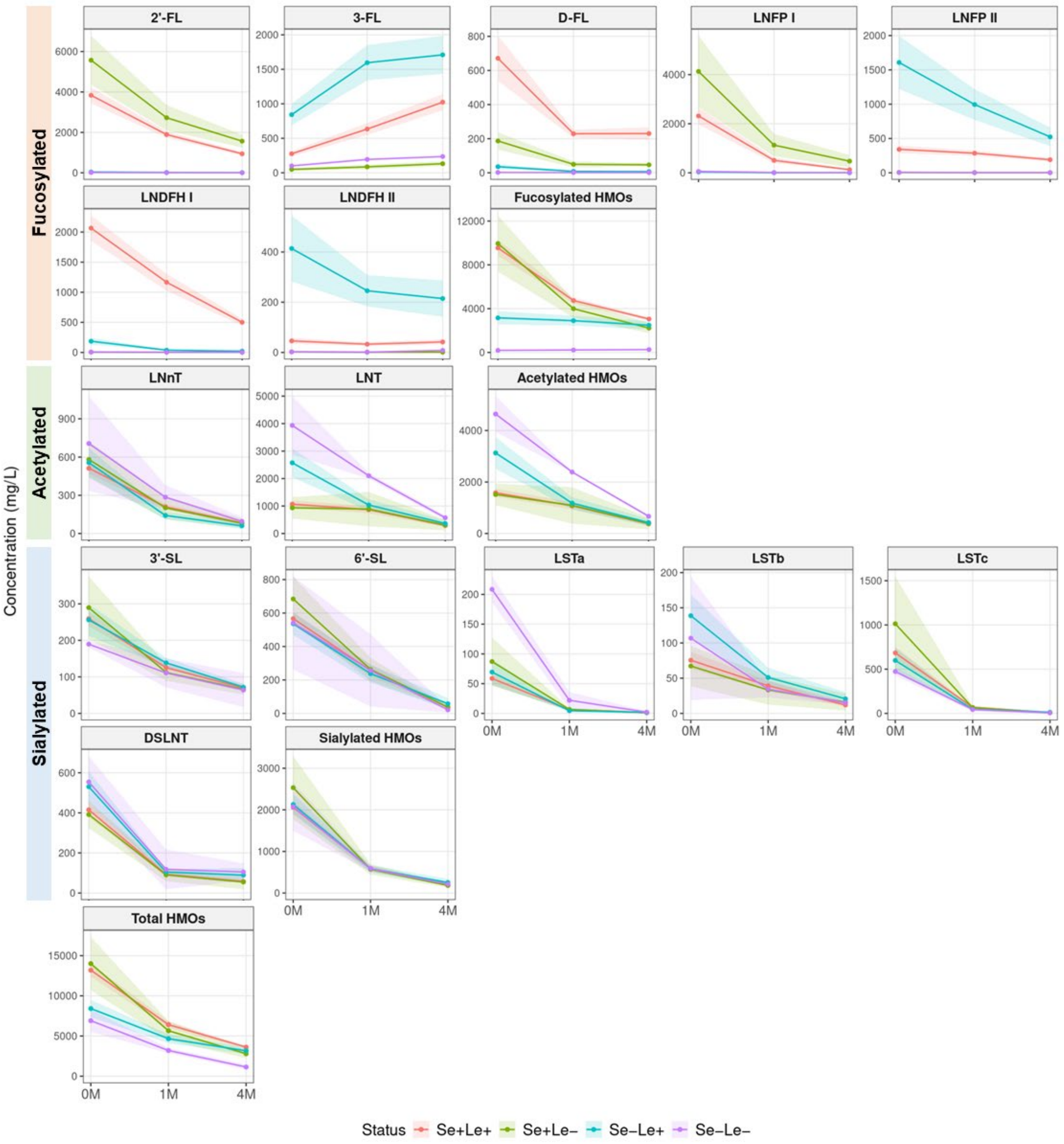

Supplementary Figure 2. Longitudinal changes of human milk oligosaccharide concentrations in human milk provided at all three time points (colostrum, 1-month milk, and 4-month milk) (n = 79) stratified according to milk groups. Colored vertical bars on the left indicate the groups based on HMO structures (fucosylated, acetylated, and sialylated). Concentrations are described by mg/L. Each dot represents the mean, and shaded areas represent the 95% confidence interval. All statistical results are shown in Supplementary Tables 6–8.

Abbreviations: 0M, colostrum; 1M, 1-month milk; 2'-FL, 2'-fucosyllactose; 3'-SL, 3'-sialyllactose; 3-FL, 3-fucosyllactose; 4M, 4-month milk; 6'-SL, 6'-sialyllactose; D-FL, difucosyllactose; DSLNT, disialyllacto-N-tetraose; HMO, human milk oligosaccharide; Le^{+}/Le^{-}, Lewis-positive/Lewis-negative; LNDFH I/II, lacto-N-difucohexaose I/II; LNFP I/II, lacto-N-fucopentaose I/II; LNnT, lacto-N-neotetraose; LNT, lacto-N-tetraose; LSTa/b/c, sialyl-lacto-N-tetraose a/b/c; Se^{+}/Se^{-}, Secretor-positive/Secretor-negative.
